# Overview on the Application of Modern Methods for the Extraction of Bioactive Compounds from Marine Macroalgae

**DOI:** 10.3390/md16100348

**Published:** 2018-09-23

**Authors:** Ana-Marija Cikoš, Stela Jokić, Drago Šubarić, Igor Jerković

**Affiliations:** 1 Faculty of Food Technology Osijek, Josip Juraj Strossmayer University of Osijek, FranjeKuhača 20, 31000 Osijek, Croatia; acikos@ptfos.hr (A.-M.C.); stela.jokic@ptfos.hr (S.J.); dsubaric@ptfos.hr (D.Š.); 2 Faculty of Chemistry and Technology, University of Split, R. Boškovića 35, 21000 Split, Croatia

**Keywords:** marine macroalgae, supercritical CO_2_ extraction, subcritical water extraction, ultrasound-assisted extraction, microwave-assisted extraction, bioactive compounds, biological activity

## Abstract

Marine macroalgae represent a rich source of bioactive compounds that can be implemented in various food, cosmetic, and pharmaceutical products for health improvement. It has been proven that these bioactive compounds, such as polyphenols, polysaccharides, carotenoids, and ω-3 fatty acids possess bioactivity. For the extraction of these compounds, modern methods (Supercritical Fluid Extraction (SFE), Subcritical Water Extraction (SWE), Ultrasound-Assisted Extraction (UAE), and Microwave-Assisted Extraction (MAE)) have been used due to their advantages over the conventional methods. The process parameters of each method must be optimized for obtaining the extracts with the targeted bioactive compounds. In distinction from the existing reviews, the present review provides novelty with respect to: (a) presenting systematically the selected process parameters of SFE (temperature, time, pressure, use of co-solvents), SWE (temperature, time, pressure, solid-solvent ratio), UAE (temperature, time, frequency, power, solid-solvent ratio), and MAE (temperature, time, frequency, power, solvent type) applied for the extractions of marine macroalgae; (b) reporting the major groups or individual compounds extracted with their biological activities (if determined); and, (c) updating available references.

## 1. Introduction

There is growing interest for new products with various bioactive compounds with potential for health improvement. It has been noticed that marine algae, except for consumption, can be used for functional products due to their bioactive compounds [1]. Our ancestors used marine algae for therapeutical purposes [2], and today they are the part of everyday diet in Asian culture. Marine algae, also called seaweeds, are divided into three classes depending on their chemical structure and pigment distribution. These classes are brown algae (*Phaeophyta*), red algae (*Rhodophyta*), and green algae (*Chlorophyta*) [3]. Red algae are the largest producers of bioactive compounds [4], which can be applied for the cosmetic, pharmaceutical, and food industry [5].

Due to the presence of biocompounds in macroalgae, they possess a wide range of bioactivities, such as anti-aging, antioxidant, antimicrobial, antiproliferative, anti-inflammatory, antidiabetic, and neuroprotective activity [5,6,7]. Since macroalgae live in extreme conditions, they must develop mechanisms of defense and learn how to adapt to these fluctuations in the environment. Because of that, macroalgae produce secondary metabolites that possess bioactivity [3].

**Figure 1:**
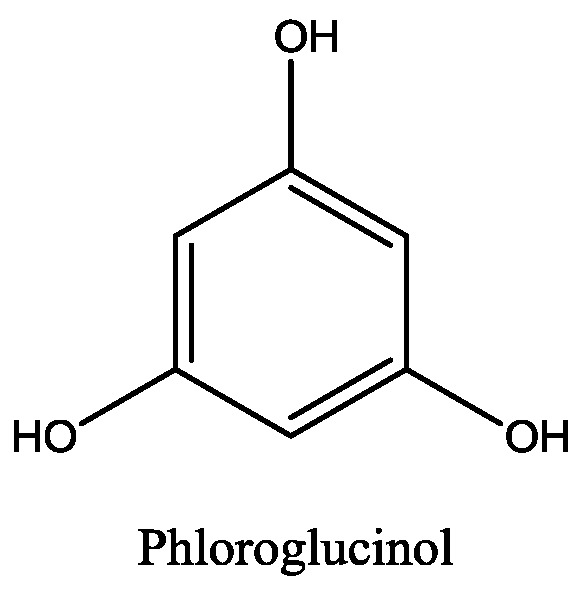
Chemical structure of phloroglucinol.

**Figure 2:**
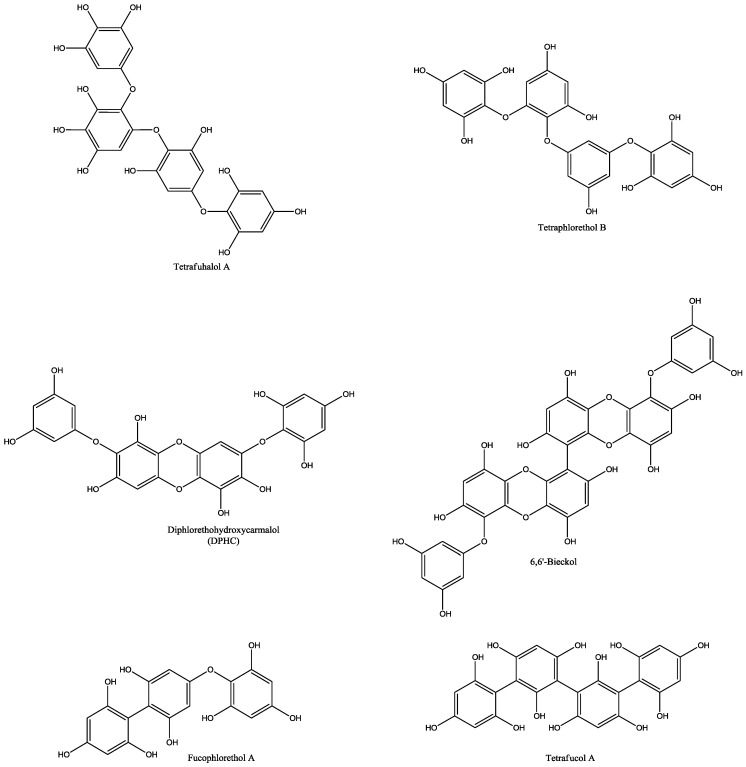
Different types of phlorotannins and their chemical structures.

The most important and researched bioactive compounds from marine macroalgae are polyphenols, polysaccharides, carotenoids, and polyunsaturated fatty acids [1]. Algae produce special type of polyphenols, named phlorotannins, which are formed of phloroglucinol units (Figure 1) [8]. Phlorotannins (Figure 2) are mainly present in brown algae and they exhibit wide range of biological activities [9]. Thomas and Kim [10] showed in the review some applications of phlorotannins and their activity e.g., anti-diabetic activity, antiproliferative activity, acetylcholinesterase inhibition activity, anti-HIV activity, and many others. 

**Figure 3:**
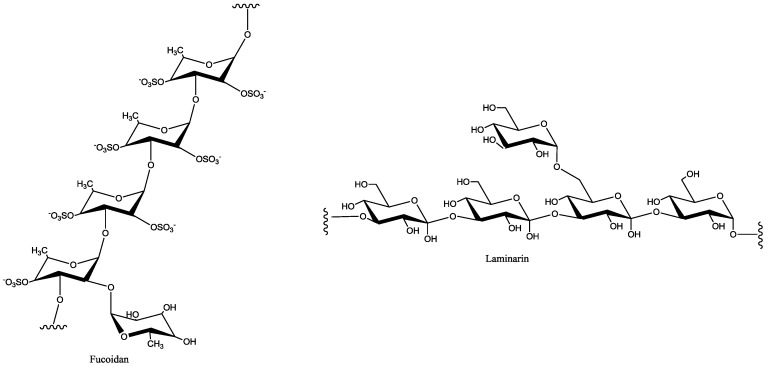
Chemical structures of polysaccharides (fucoidan and laminarin).

Algae are considered as a good source of dietary fibers because of the presence of non-digestible polysaccharides in the algal cell wall (Figure 3) [11]. It has been shown that they possess activities, like antioxidant [12], hypoglycemic [13], antiviral [14,15], antitumor [16], and anti-inflammatory due to the presence of fucoidan and laminarin [17]. 

**Figure 4:**
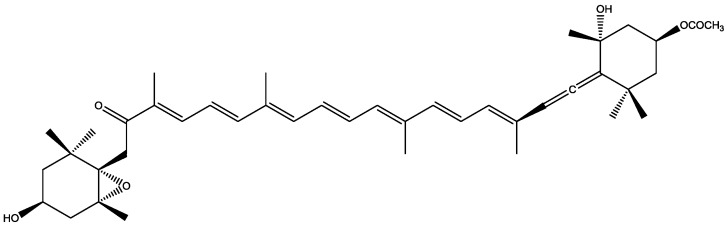
Chemical structure of fucoxanthin.

Algae are known photosynthetic organisms that contain pigments, like chlorophylls and carotenoids. Carotenoids are natural antioxidants and they are applied in different food products [18]. The most abundant carotenoid of marine macroalgae is fucoxanthin (Figure 4) and it possess antiproliferative activity [19,20]. Except for antiproliferative properties, carotenoids also possess antioxidant [21], antimicrobial, and antihypertensive activity [22].

**Figure 5:**
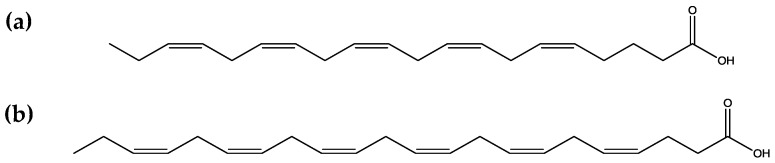
Chemical structure of eicosapentaenoic acid (EPA) (**a**) and docosahexaenoic acid (DHA) (**b**).

Since polyunsaturated fatty acids (Figure 5) show biological properties, like cardiovascular protection, antiproliferative, and anti-inflammatory activity, their presence in macroalgae makes them very important for human health [6]. Macroalgae contain larger amount of unsaturated fatty acids than saturated [23,24], and because of that, they exhibit acetylcholinesterase inhibition, which means that they can provide protection from neurodegenerative disorders [25].

The extraction of these compounds can be performed by conventional methods or new alternative methods. Alternatives, often classified as green methods, showed several benefits over the conventional methods, including reduced amounts of used solvent, shorter extraction time, and performance at lower temperature. These methods have better selectivity for the isolation of desired compounds, while the formation of byproducts and unwanted reactions during the extraction are avoided [26,27]. As a result of large differences in the structure among the different classes of target bioactive compounds and their natural sources, their physical and chemical properties differ. Therefore, it is very important and necessary to find the most efficient method of the extraction of selected bioactive compounds and then optimize the extraction procedure. Innovative extraction techniques (without the use of enzymes), which are mostly applied in the isolation of bioactive compounds from marine macroalgae, are Supercritical Fluid Extraction (SFE), Subcritical Water Extraction (SWE), Ultrasound-Assisted Extraction (UAE), and Microwave-Assisted Extraction (MAE). It is necessary to examine the process parameters of each extraction procedure to obtain a true insight about the impact of particular method on the content of the bioactive compounds in the obtained extracts.

Several reviews on this topic are available reporting: conventional and novel extraction methods from algae [28,29], as well as pre-treatment of algal biomass [28], the extraction of bioactive compounds from other marine organisms, such as bacteria, diatoms, sponges, crustaceans, mollusca, echinodermsand fishes [30], the extraction methods with an emphasis on the sub- and supercritical fluids [31], and the usage of SFE for extraction of ω-3 fatty acids from fishes and algae [32]. Those reviews showed the application of novel methods for extraction of bioactive compounds from both microalgae and macroalgae, but either of these reviews provided information only for one type of marine algae. However, microalgae were more mentioned than macroalgae because there are more available data in the literature about their bioactive compounds, as well as their potential for being implemented into industry, for example, due to their ability to store high amounts of long-chain hydrocarbons they can be used as substitutes of natural waxes [29,31]. Therefore, the target of the present review was to update the information of macroalgae and their potential for implementation in various products. Consequently, the emphasis was on the macroalgae and their bioactives that were extracted with the most applied innovative techniques (without the use of enzymes), such as SFE, UAE, SWE, and MAE. Also, neither of mentioned reviews observed detailed behaviour of the process parameters of each extraction method and their influence on the chemical composition of the extract and the possibility of obtaining the desired compounds by changing the conditions and parameters of extraction. However, the present review provides novelty with respect to: (a) presenting systematically the applied process parameters for the modern extractions (SFE, UAE, SWE, and MAE) to obtain the desired compounds from marine macroalgae; (b) reporting the major groups or individual compounds extracted with their biological activities (if determined); and, (c) updating available references.

## 2. Novel Extraction Techniques of Bioactive Compounds from Marine Macroalgae

### 2.1. Supercritical Fluid Extraction (SFE)

SFE is based on the principle of extraction with fluids in their supercritical conditions, temperature, and pressure are raised above their critical point with characteristics of both liquids and gases [33]. The fluid density is similar to the values found for liquids, while its viscosity is close to values of gas [34]. Carbon dioxide (CO_2_) is the most used solvent for SFE due to its nontoxicity, safety, and low cost [33]. Major advantage of supercritical fluid is increased mass transfer due to low viscosity and higher diffusion coefficient. Supercritical CO_2_ (SC-CO_2_) can extract only nonpolar or compounds of low polarity since it is nonpolar solvent but the extraction of polar compounds can be enhanced by adding small amounts of polar co-solvents such as ethanol or methanol [27]. Conditions during the extraction, especially pressure and temperature, are responsible for selectivity and solubility of the various compounds in the supercritical fluid [20]. CO_2_ has low critical temperature and pressure, which means that bioactive compounds stay preserved and no degradative changes can occur [35]. Usually the extracts, obtained with SC-CO_2_, contain groups of compounds, like fatty acids, phytosterols, tocopherols, phenolics, carotenoids, and triglycerides [34].

To date, most of the published reports of SC-CO_2_ from marine macroalgae were directed toward the isolation of phenolic compounds and carotenoids. Biological activity of isolated compounds was determined and reported by many researchers [21,22,36,37]. Regarding the gathered information in Table 1, the authors applied various ranges of pressures and temperatures to obtain desirable bioactive compound. SC-CO_2_ was used mostly for the extraction of polyphenols and carotenoids where applied pressures were at range mostly of 20–30 MPa and some of the authors used ethanol (EtOH) as co-solvent [21,22,37,38], whereas, temperature was applied in the range 40–60 °C. The extraction efficiency of phenolic compounds and carotenoids increased when EtOH is used as co-solvent when compared to the use of SC-CO_2_ without co-solvent [21,22]. Ospina et al. [37] noticed that the amount of extracted polyphenols increased with increasing the polarity and density of CO_2_ when EtOH was used as co-solvent due to dipole-dipole interactions and formation of hydrogen bonds. Saravana et al. [39], except of using EtOH as co-solvent, applied water and various types of oils, such as sunflower, soybean, and canola oil to compare their effectiveness for improving the extraction of total carotenoids, fucoxanthin, and phlorotannins. When sunflower oil was used as co-solvent, the highest yield of total carotenoids and fucoxanthin was obtained, while for phlorotannins, water was the best co-solvent. However, the extraction yield of bioactive compounds is in relation with process parameters that have the influence on SC-CO_2_ density and vapor pressure of the compounds [38]. As the pressure increases, the extraction yield also increases [40] due to increased density and solvating power of SC-CO_2_ [41]. On the other hand, the influence of temperature on the extraction yield is more complex. Therefore, retrograde behavior occurs, meaning that an increase in temperature at low pressure exhibited a negative effect on the extraction yield and a positive effect at high pressure [40]. Most of the reported studies have shown that highest yield of carotenoids was at pressure of 30 MPa and temperature of 50 °C [20,21,38], whereas, Quitain et al. [42] showed that the highest yield of fucoxanthin was obtained at a pressure of 40 MPa and temperature of 40 °C, and with the increase of temperature the yield of fucoxanthin decreased due to the degradation of fucoxanthin and decreased solubility in SC-CO_2_. Fucoxanthin is one of the most abundant marine carotenoids present in brown algae, but studies have shown that it also can be present in green algae from biotransformations of different carotenoids [20,21]. Roh et al. [38] observed that the extraction of polyphenols increased with increasing the pressure and temperature. The latter was explained with the fact that polyphenol vapor pressure was dominant in solvating power. Tanniou et al. [36] compared phenolic profiles among the extracts that were obtained with various extraction techniques and noticed that the extracts that were obtained with SFE showed very different phenolic profile to those obtained with the other methods. The solvent used for the extraction have significant effect on phenolic extraction due to its polarity. Besides, macroalgae species as well as the season of their collecting affects phenolics extraction. It is important to collect macroalgae during the summer [36] because then they produce the maximum of phenolic compounds. Sivangnanam et al. [22] obtained the extracts of *S. japonica* and *S. horneri* with SC-CO_2_ with ethanol as co-solvent, which showed significant antioxidant activity. Hence, total phenolic content is also determined because phenols are the main contributors to the antioxidant activity, and it was shown that brown seaweeds have a higher concentration of polyphenols than red seaweeds. Except antioxidant activity, antimicrobial and antihypertensive activity of the extracts were determined. The extracts showed good antimicrobial activity against *Listeria monocytogenes*, *Bacillus cereus*, *Staphylococcus aureus, Escherichia coli, Candida albicans,* and *Aspergillus brasiliensis.* Moderate antihypertensive activity was noticed for the extracts of *S. japonica* and *S. horneri,* where fucoxanthin was mostly involved. Ospina et al. [37] applied obtained extracts for the protection of an edible oil to evaluate antioxidant activity of the extracts. The results showed that the extracts from red seaweed *Gracillaria mammillaris* protected the oil from the lipid oxidation, but its activity was lower than synthetic antioxidants. 

It can be noticed that most of the studies were directed toward the extraction of polyphenols and carotenoids. However, some authors used SC-CO_2_ for the extraction of lipids [41], volatile compounds [43], isoflavones [44], plant growth hormones, and micro- and macroelements from various types of seaweeds [45]. Hattab et al. [43] analyzed the volatile compounds from the extracts and they observed the presence of sesquiterpenes, C_11_-hydrocarbons and sulphur compounds, the latter being most present with main compound 3-hexyl-4,5-dithiacycloheptanone. Michalak et al. [45] applied the extracts for observation of growth stimulant activity of wheat and cress. The results showed that algae extracts stimulated the growth of the parts above the ground of both wheat and cress. Consequently, chlorophyll and carotenoid content also increased due to enhanced growth of parts that were responsible for the photosynthesis process, which are above the ground. Cheung [41] analyzed the influence of pressure and temperature on the extraction yield of lipids and fatty acid content. Generally, algae contain low amounts of lipids, but they are rich in ω-3 fatty acids, such as α-linolenic (ALA, 18:3) and eicosapentaenoic acid (EPA, 20:5), while docosapentaenoic acid (DPA, 22:5) and docosahexaenoic acid (DHA, 22:6) are present in lower concentrations. Also, unsaturated fatty acids (including ω-3 fatty acids) comprise around 60 % of the total fatty acids, which are found in seaweeds. Seaweed lipids, especially ω-3 polyunsaturated fatty acids, exhibit several advantages for human health, such as the prevention of cardiovascular diseases, diabetes, hypertension and autoimmune diseases, and even for the prevention of arterosclerosis [22].

### 2.2. Ultrasound-Assisted Extraction (UAE)

UAE uses ultrasound waves with a frequency above 20 kHz to 100 kHz. These waves cause the creation of bubbles and zones of high and low pressure. When bubbles collapse in the strong ultrasound field cavitation occurs. The implosive collapse, cavitation, near liquid-solid interfaces causes breakdown of particles, which means that mass transfer is increased and bioactive compounds are released from biological matrix [29]. Ultrasound equipment can be ultrasonic bath (indirect sonification) or ultrasonic probe (direct sonification). The differences between these two are operating conditions and the way the ultrasound waves affect the sample. Ultrasonic bath operates at frequency of 40–50 kHz and at power of 50–500 W, but ultrasonic probe can operate only by the frequency of 20 kHz. The samples are immersed in the ultrasonic bath, whereas, the ultrasonic probe is inserted into the sample [47]. Costs of the equipment are lower than the other alternative extraction techniques and wide variety of solvents can be used [29]. UAE operates with low temperatures which enables preservation of thermolabile compounds and prevents completely damage of the structure [48]. Low amounts of solvent are used and the working time of extraction is reduced, which makes UAE a fast, inexpensive method as compared to traditional methods [33].

Available data of UAE extraction of macroalgae shows that polyphenols and antioxidants are the main extracted compounds from macroalgae (Table 2). Various solvents were used, such as ethanol, distilled water, and methanol with different solid:solvent ratio [7,49,50]. 

Dang et al. [49] optimized the ultrasonic conditions for obtaining the highest yield of total phenolic content (TPC), and, consequently, higher antioxidant activity. They concluded that optimum conditions were temperature of 30 °C, time of 60 min and power of 60% (150 W). Topuz et al. [51] obtained a higher content of polyphenols with process parameters of 50 °C, time of 45 min, and solvent:seaweed ratio 30:1 mL/g, but with higher power of ultrasonic bath of 250 W. Each of these parameters affected the extraction efficacy of polyphenols. Increasing the extraction temperature, the extraction yield increased due to higher mass transfer and solvent diffusion rate. Also, the extraction time had to be optimized for the prevention of degradation of phenolic compounds and for the increase of extraction efficacy. The higher ultrasonic power lead to an increase in TPC because the cell wall was more damaged and the solvent could easily penetrate into solid material. According to Dang et al. [49] the temperature exhibited the strongest influence on the extraction yield and amount of extracted phenolic compounds, while Topuz et al. [51] concluded that the solvent:seaweed ratio exhibited the most influence on TPC. But, it must be taken into consideration that authors used different species of macroalgae, and this can be the reason why the results differ. Also, these studies showed that antioxidant activity increased as TPC increased because phenolic compounds contribute most to the antioxidant activity. However, antioxidant activity differs between seaweed species and extracts due to biological variation making difficulties for the comparison. The higher amount of TPC is shown by Kadam et al. [52], because they used acid (0.03 M HCl) as solvent, while Dang et al. [49] used ethanol. But, both authors used various species of macroalgae, which explained the difference between amounts of extracted polyphenols. Except the extraction of phenols, Kadam et al. [52] also observed the extraction of fucose and uronic acid. They showed that the higher extraction yield was obtained for phenolic content and uronic acid when acid was used, while the use of acid did not contribute to the higher fucose content. If the higher concentration of acid was used, the decrease in the extraction yield occurred due to acid hydrolysis and degradation effects. Analyzed extracts showed the presence of higher molecular weight phlorotannins with acid as solvent when compared to the use of water as solvent. Similar results were reported for the UAE of high molecular weight laminarins. UAE, with acid (0.03 M HCl) as solvent, has been successfully used to obtain laminarin in the extracts. These extracts contained higher laminarin content than the extracts where water was used as solvent, and antioxidant activity was higher in those extracts. The antimicrobial activity of the extracts was determined for the inhibition of *Staphylcoccus aureus, Listeria monocytogenes, Escherichia coli* and *Salmonella typhimurium.* The extracts that were obtained with acid showed better inhibition of bacterial growth when compared to the extracts that were obtained with water [53]. Lee et al. [50] conducted much longer time of the UAE unlike other authors [49,52]. They compared the yield and the antioxidant activity of UAE extracts with the extracts that were obtained with conventional method (CE). The extracts obtained with UAE showed higher yield than those of CE. The higher amount of TPC was in the extracts obtained with CE and the results showed that the yield and TPC are time-dependent. UAE extracts showed good radical scavenging activity and an inhibitory effect against DNA damage induced by H_2_O_2_. 

Cell-wall disruption is required for the efficient release of molecules during UAE. As mentioned above, cavitation occurs and it results in the disruption of cell-wall. As phycobiliproteins are intracellular molecules and their extraction is difficult due to the presence of large polysaccharides, UAE must be combined with some conventional methods, such as maceration and homogenization. Mittal et al. [54] showed that a combination of maceration and ultrasonication resulted in the highest yield and efficiency of extracting phycobiliproteins. Anyhow, the process parameters must be precisely determined. For instance, ultrasonication time can result in an increase in temperature, which is not desirable for stability of phycobiliproteins. Similar as Mittal et al. [54], Rodrigues et al. [7] concluded that polysaccharides can interfere the extraction of molecules through cell wall due to their complexity and amount, so the fundamental step is degradation of their structure, which leads to the release of the compounds from seaweeds. They also reported that antioxidant activity varies among the species and applied extraction method. It was shown that brown algae had higher phenolic content and antioxidant activity than red and green algae. However, the radical scavenging activity of phenolic compounds depends on their structure, number and location of hydroxyl groups. Also, authors applied seaweed extracts to observe prebiotic potential of *Lactobacillus acidophilus* and *Bifidobacterium animalis*. Results showed that the seaweed extracts possess carbon sources that can be metabolized by mentioned bacteria. The carbon sources are mostly polysaccharides, but not agar, because microorganisms are not able to hydrolyze and metabolize it. Wang et al. [55] used UAE for the extraction of taurine, which has numerous beneficial effects, such as protecting the liver and lowering blood pressure. According to the results, UAE can be applied for the extraction of taurine with optimal parameters of 40.5 °C, 38.3 min, and 300 W. When compared to the conventional solid-liquid method, UAE provided higher yield of taurine and less extraction time was needed. The authors analyzed taurine contents before and after sonication to evaluate the degradation of taurine during UAE. No significant changes in taurine content occurred, so UAE was successful for obtaining taurine without degradation.

### 2.3. Subcritical Water Extraction (SWE)

SWE operates at high temperatures (50–200 °C) and pressures (50–300 psi) for a short period of time (5–10 min) with a small amount of solvent. According to published studies [14,31,36], this is the most promising technique for the extraction of bioactive compounds. The solvents are maintained near their critical-region in the liquid state with the help of applied temperature and pressure, keeping the solvents below their boiling point [33]. Increasing the operating temperature, solubility, and mass transfer rate are enhanced due to decreased viscosity and surface tension of the solvent. SWE is environmentally friendly extraction because of water, which is used as a solvent instead of using the organic solvents. Physical and chemical properties of water are changed during the extraction because high temperature and pressure are applied. Consequently, the dielectric constant of water is significantly decreased from 80 (25 °C) to 33 (200 °C), which is close to the dielectric constant of methanol [56]. It can be concluded that SWE can be used for extracting nonpolar compounds by replacing some organic solvents. In addition, it offers higher extraction yields because the permeability of solvent into the material is enhanced and there is no influence on the extracted bioactive compounds. But, the extraction time must be controlled because degradation of compounds may occur [57].

Various conditions of SWE were shown in the available data (Table 3) and optimal conditions for each extracted compound were determined. The extracts and bioactive compounds have shown bioactivity such as antioxidant, antiviral, antimicrobial and anti-hyaluronidase activity [14,34,58,59]. 

del Pilar Sanchez-Camargo et al. [34] demonstrated the application of SWE with enzymes as pre-treatment for increasing the extraction yield and phlorotannins recovery, but it did not gave the best results. Usage of SWE without the enzymes showed to be an efficient method for obtaining polyphenols and phlorotannins. However, when using water as solvent the highest yield was obtained, but total phenols content and total phlorotannins were lower when compared to ethanol. Ethanol in subcritical conditions was more selective toward the extraction of polyphenols and phlorotannins and these extracts showed higher antioxidant activity. Authors tested various extraction methods to obtain rich antioxidant extracts that can be applied for functional foods or as ingredients to reduce or inhibit oxidative deterioration of foods [60,61,62]. These natural antioxidants can replace synthetic antioxidants which have been added to many foods but there is a growing concern about their toxicity and safety for human health [63]. Phlorotannins exhibit the most potential for being used as natural antioxidants due to their condensed structure with the ability to scavenge free radicals from multiple sites. Heffernan et al. [64] investigated two brown algae, one red and one green alga. Their results showed that brown algae contained the highest amount of polyphenols, and, consequently, the highest antioxidant activity due to correlation between TPC and antioxidant properties. Brown macroalgae generally have higher content of polyphenols than red and green macroalgae, due to the presence of phlorotannins, which were not present in the other macroalgae groups [65]. While Tierney et al. [66] in their research showed that the extracts obtained with conventional method contained higher concentration of extracted polyphenols compared with SWE extracts, Vo Dinh et al. [67] obtained the opposite results. When dielectric constant decreases with an increasing temperature and pressure during SWE, a higher amount of polyphenols were extracted. The explanation for the extraction of a lower amount of polyphenols might be linked to the loss of thermally labile compounds. The extraction temperature seems to have the highest influence on the yield of polyphenols and on the antioxidant activity. Vo Dinh et al. [67] noticed that phenolic content increased as the temperature rise from 100 °C to 225 °C, but when it reached 250 °C, the phenolic content started to reduce. The same behavior is noticed for the antioxidant activity of the extracts, which showed that phenolic content and antioxidant activity are correlated.

Regarding to capacity of restoring the calcium metabolism in epidermis and anti-age effect of the skin, brown alga *Padina pavonica* can be used for anti-hyaluronidase activity and it can be implemented for cosmetic use. Fayad et al. [58] used capillary electrophoresis-based enzymatic assays for the evaluation of the activity towards hyaluronidase of *Padina pavonica* extracts obtained by SWE. Results showed that water extract obtained by SWE yielded the most potent inhibition when compared to other extraction methods such as MAE, SFE and pressurized liquid extraction (PLE). Further analysis of the extract is needed to characterize and identify the molecules that are responsible for hyaluronidase inhibition. It is known that water mainly extracts sulfated polysaccharides, which are known for their anti-hyaluronidase activity and also for antiviral and antioxidant activity.

During SWE, some compounds, such as thermolabile compounds, can be degraded because of the high temperatures that are applied [64]. On the other hand, some compounds can be formed due to the reactions that occur during the extraction. For example, products of Maillard reactions or caramelization, which can be desired in some cases. Except mentioned reactions, the increase in the antioxidant activity when temperature is increased can be explained with the formation of new antioxidants as a result of interaction between the compounds [68]. Plaza et al. [69] used six different species of macroalgae and all of them were extracted with the same conditions. In all cases, samples that were obtained at higher temperature exhibited higher antioxidant capacity than those that were obtained at lower temperature. This phenomenon can be explained by the formation of neoantioxidants which are products of several chemical reactions including Maillard and caramelization reactions. Saravana et al. [70] measured absorbance to check the development of browning reactions during Maillard reactions. It was shown that, as the extraction temperature increased, the absorbance was higher and it indicated the advanced formation of brown products of Maillard reactions and caramelization. These products are applied in the food and beverages and they have significant importance for nutritional value. Except for positive effects, like strong antioxidant activity, some compounds, such as hydroxymethylfurfural, can be toxic. 

Polysaccharides, including fucoidan, laminarin, and alginates, exhibit antiviral and antioxidant properties. The antiviral activity of polysaccharides is based on their ability to interfere with the initial attachment of the virus and blocking entry of the virus to the cell. Santoyo et al. [14] examined the extracts obtained by SWE against *Herpes simplex* virus type 1. After that, the extracts were analyzed by GC-MS, and it was shown that they consist mostly of polysaccharides. These compounds exhibited antiviral activity when the extracts were added previously to virus or simultaneously with virus. Crude fucoidan was examined for antioxidant activity by Saravana et al. [12], and it was considered that some other compounds contribute to antioxidant activity. It was assumed that the presence of hydrogen atoms from specific monosaccharide compositions and side-chain linkages of polysaccharides contribute to the ability of radical scavenging.

### 2.4. Microwave-Assisted Extraction (MAE)

MAE is based on ionic conduction and dipole rotation which act directly on the molecules and occur simultaneously. Microwave heating causes absorption of energy by molecules where no heat is lost into the environment. Due to absorption of energy by polar molecules, disruption of cells is inevitable. Destructed cells facilitate faster mass transfer and diffusion out of solid, where mass and heat transfer act synergistically and in the same direction [71]. MAE can be performed in open or closed vessels. Open vessels operate at atmospheric pressure, while closed vessels operate at pressure that is higher than atmospheric. Due to operation at atmospheric pressure, open vessels can be more effective, safer, and it is possible to process larger samples. Also, process conditions are suitable for thermolabile compounds [72]. An advantage of MAE is that it is economical and environmentally friendly process because of the reduced process time and solvent amount [73].

The most microwave-assisted extracted compounds from macroalgae are polyphenols and polysaccharides (Table 4). Authors optimized the extraction conditions such as power and frequency of microwaves, solid:solvent ratio, temperature and time to obtain higher extraction yields and better isolation of these compounds [60,74,75].

When extracting polyphenols, the microwave power plays the key role in the extraction yield, according to Li et al. [60]. If power is too high, degradation of the phenolic compounds occurred. Similar behavior is noticed in the case of the extraction temperature. The effect of ethanol concentration as a solvent in MAE also must be considered. Even though, water can efficiently absorb microwave energy, which leads to evenly heating, ethanol concentration contributes to the solubility of phenolic compounds. But, if ethanol content in the extraction medium was too high, the yield of extracted polyphenols decreased. It was explained with water as the polar solvent and the principle of “like dissolves like”. Hence, the antioxidant activity of the extracts was determined and it was shown that the extracts with the highest total polyphenol content exhibited the highest antioxidant activity. The results, which are shown by Lou et al. [76], vary slightly in the terms of extraction conditions and their influence on the extracted polyphenols. In this case, the highest influence on the yield of phenolic compounds was from the extraction cycles. They also consider the effect of time on the extraction yield of polyphenols and showed that, if the material is over exposed to the microwaves, degradation of polyphenols can occur. The polyphenols content vary among the species of algae because it depends on their habitat and environmental conditions. Magnusson et al. [74] screened 100 species of brown algae that were collected from different areas and their polyphenols content was examined. Results showed that the content varied not only at taxonomic and geographical levels, but also within each of these levels between orders of algae. Except the taxonomical differences, season time and processing methods (such as drying) also affected the concentration of polyphenols. Polyphenols are present as structural elements of the cell wall or as secondary metabolites present in cytoplasmic physodes. Authors noticed that water, as green solvent, has been the most suitable solvent for efficient extraction of polyphenols when compared to other tested solvents and also that the extraction yield of polyphenols that were obtained by MAE grew up to 70% when compared to solid-liquid extraction with organic solvents. Zhang et al. [77] were the first who compared antioxidant activity of crude algal extracts with ascorbic acid and their thermal stability to investigate possibility of replacing ascorbic acid as natural antioxidant in food products. The results showed that crude algal extracts were much more stable than ascorbic acid and they could be applied as natural antioxidants.

Except antioxidant activity, the extracts obtained by MAE also showed anti-hyaluronidase activity. Fayad et al. [58] conducted short time of the extraction with various solvents, each of which gave the best inhibition results at different extraction temperatures. The hyaluronidase inhibition is highly influenced by the extraction temperature rather than the extraction time, because increasing the temperature, solubility, diffusion rate, and mass transfer are improved, and, at the same time, the surface tension and viscosity of the solvent are decreased. According to the results, water and ethyl acetate showed the best inhibition results, which indicates that anti-hyaluronidase bioactive compounds are mostly polar. 

Algae contain a larger amount of polysaccharides during winter because they generate reserve during the spring in their rapid grow phase in order to survive winter when photosynthesis could not occur. According to Rodriguez-Jasso et al. [78] the pressure was the main parameter influencing the fucoidan composition. Low pressure exhibited less destructive effect of algae structure which means that less polysaccharides can be released from the cells. Compositional characterization of the extracted fucoidan showed that the conditions of MAE affected sulfating degree of fucoidan. It is important to obtain high sulfate content because they possess biological functions such as anti-HIV activity. Ren et al. [75] showed that the extracts with polysaccharides exhibited good inhibitory effects against α-glucosidase, and they could be potential hypoglycemic agent applied in food or pharmaceutical industry. Similar as on the previous mentioned polyphenols content, the extraction conditions have the influence on the polysaccharides content. As time increased, the yield also increased, but excessive time can have the opposite effect and it can lead to degradation of polysaccharides. Similar behavior occurred during the increase of microwave power and the extraction temperature. Quitain et al. [79] observed how MAE influenced the degradation of fucoidan on low molecular weight compounds. These compounds are more valuable because the data available in the literature reported that some biological activities, such as antiproliferative activity of fucoidan, depend on the molecular weight of fucoidan fractions. Low molecular weight products of polysaccharides showed higher antiproliferative activity and those with higher sulfate content exhibited strong antioxidant activity [80,81]. Polysaccharides have shown pancreatic lipase inhibition activity in all tested extracts. It was suggested that the explanation of such activity is attributed to the antioxidant potential of polysaccharides. In their study, Yuan et al. [82] showed that sulfur content had more significant influence on the antioxidant activity rather than molecular weight of polysaccharides. The increased temperature affected the sulfate content of fucoidan, it decreased with the extraction temperature [81]. When compared to the conventional extraction, MAE could faster obtain the extracts with low molecular weight products of degraded fucoidan. During the microwave irradiation with hydrothermal heating, the degradation of fucoidan was enhanced due to the thermal effects such as molecular agitation, localized heating and improved mass transfer [79]. MAE has also been good method for the extraction of ulvan and rhamnan sulfate, since it does not require toxic solvents and these compounds can be applied for food and biomedical purposes. Both of the compounds exhibit biological activities, such as antitumor, anticoagulant, antiviral, and antiherpetic [83]. Yuan et al. [82] were the first ones that reported water-holding (WHC) and oil-holding capacity (OHC) and also foaming properties of algae polysaccharides. These functional properties make algae polysaccharides potential for appliance into food to modify texture, stabilize emulsions, and used as thickeners. Authors showed that all three properties are dependent of the molecular weight of polysaccharides. Lower molecular weight showed better WHC, OHC and foaming properties. Furthermore, they concluded that algae polysaccharides can be used in different industrial application due to extremely good functional properties.

## 3. Conclusions

Macroalgae contain various bioactive compounds with application in the food, cosmetic, and pharmaceutical industry. They showed potential for developing of new functional products which can have positive influence on human health. The alternative modern green extraction techniques which were presented in this review showed their potential for implementation in the industry for the isolation of bioactive compounds from marine macroalgae. Green, red, and brown macroalgae showed significant differences in their chemical composition and each of these algae have great potential for different products. SFE has been good method for obtaining the extracts with fatty acids and lipids, including carotenoids, such as fucoxanthin. UAE is mostly used for the extraction of polyphenols and their correlation with antioxidant capacity of macroalgae is given. Water and ethanol were shown as the best solvents for UAE of polyphenols. Water has been successful solvent also under the subcritical conditions in SWE. The most analyzed compounds in SWE extracts were polyphenols. Authors established that during SWE, new antioxidants were formed and they contribute to the antioxidant capacity of the extracts. Formation of new antioxidants is the result of Maillard reactions and caramelization which occurred during the extraction process under the subcritical conditions. MAE is the most studied extraction process and successful for isolation of bioactive compounds from marine macroalgae and obtained extracts are rich in sulfated polysaccharides, such as fucoidan, ulvan, and rhamnan sulfate, which exhibit antioxidant, anti-hyperlipidemic, and hypoglycemic activity. Also, polyphenols, especially phlorotannins from brown algae, have been shown to possess strong antioxidant activity.

Regarding to the given information, it can be concluded that methods, such as SFE, UAE, SWE, and MAE can be applied for the isolation of specific bioactive compounds. Optimizing their process parameters, desired extraction yield, and chemical composition of the extracts can be achieved. The main advantage is that the extraction of targeted compounds and their solubility can be controlled by applied process parameters of each method. On each of the above mentioned extraction methods, different process parameters showed direct influence on the extracted bioactive compounds. The presented data about macroalgae contributed updating information of great potential of these marine organisms. Due to their biological activity, application in the functional products and the influence on human health are inevitable. Preserving sensitive compounds by using novel methods that are mentioned in the present review makes them suitable for producing the extracts of higher value. 

## Figures and Tables

**Table 1:** Supercritical CO_2_ (SC-CO_2_) extraction of bioactive compounds from marine macroalgae and their bioactivity.

Macroalgae Species	Extraction Parameters				Bioactive Compounds	Bioactivity	Ref.
	Pressure [MPa]	Temp. [°C]	Time [min]	Co-Solvent [%]			
*Hypnea charoides*	24.1–37.9	40–50	120	-	Fatty acids (ω-3)	-	[41]
*Cladophora glomerata, Ulva flexuosa, Chara fragilis*	10–30	40–60	120	EtOH [0–15]	Carotenoids, phenols	Antioxidant	[21]
*Dictyopteris membranacea*	9.1 and 10.4	40	30	-	Volatile compounds	-	[43]
*Fucus serratus, Laminaria digitata*	15, 22.5 and 30	30, 40 and 50	60 and 105	-	Carotenoids	-	[20]
*Sargassum muticum, Sargassum vulgare, Hypnea spinella, Porphyra* sp*., Undaria pinnatifida, Chondrus crispus, Halopytis incurvus*	10–40	35–75	10–60	-	Isoflavones	-	[44]
*Cladophora glomerata, Ulva flexuosa subsp. pilifera, Ulva clathrata, Polysiphoniucoides*	50	40	300, 360, 810	-	Polyphenols, cytokinins, auxins, microelements and macroelements	Plant growth stimulation	[45]
*Gracilaria mammillaris*	10,20 and 30	40, 50 and 60	240	EtOH [2,5,8]	Polyphenols, carotenes	Antioxidant	[37]
*Undaria pinnatifida*	22.9	45	50	-	Hydrocarbons	-	[46]
*Undaria pinnatifida*	20–40	25–60	180	-	Fucoxanthin	-	[42]
*Undaria pinnatifida*	8–30	30–60	50	EtOH [3]	Fucoxanthin, polyphenols	-	[38]
*Saccharina japonica (Laminaria japonica)*	20–30	45–55	240	Sunflower oil, soybean oil, canola oil, EtOH and water [0.50–2.00]	Carotenoids, fucoxanthin, phlorotannins	Antioxidant	[39]
*Saccharina japonica, Sargassum horneri*	25	45	120	EtOH	Fatty acids, fucoxanthin, polyphenols	Anti-oxidant, anti-microbial and antihyper-tensive	[22]

**Table 2:** Ultrasound-Assisted Extraction (UAE) of bioactive compounds from marine macroalgae and their bioactivity.

Macroalgae Species	Ultrasound Operating Conditions					Bioactive Compounds	Bioactivity	Ref.
	Ultrasound Equipment; Frequency [kHz]; Power [W]	Sample Mass [g]	Solvent Volume [mL]	Temp. [°C]	Time [min]			
*Hormosira banksii*	Ultrasonic bath; 50; 150–250	1	50 (70% ethanol)	30, 40 and 50	20, 40 and 60	Polyphenols	Antioxidant	[49]
*Ascophyllum nodosum*	Ultrasound probe; 20; 750	4	40 (distilled water and 0.03 M HCl)	-	10	Polyphenols, fucose and uronic acid	-	[52]
*Ascophyllum nodosum, Laminaria hyperborea*	Ultrasound probe; 20; 750	10	200 (distilled water and 0.03 M HCl)	-	15	Polyphenols, laminarin	Antioxidant	[53]
*Ecklonia cava*	Ultrasonic bath; 40; 200	1	100 (water; 50% methanol; 100% methanol)	30	360 and 720	Polyphenols	Antioxidant	[50]
*Gelidium pusillum*	Ultrasonic bath; - 41.97	10	100 (phosphate buffer 0.1 M)	30, 35 and 40	2, 4, 6, 8 and 10	Phycobili-proteins	-	[54]
*Sargassum muticum, Osmundea pinnatifida, Codium tomentosum*	Ultrasonic bath; 50/60; 400	2	50 (deionized water)	50	60	Total phenolics, antioxidants, prebiotic compounds	Antioxidant, prebiotic, α-glucosidase inhibition	[7]
*Laurencia obtuse*	Ultrasonic bath; 40; 250	1	10–30 (95% ethanol)	30–50	30–60	Phenolic compounds, antioxidants	Antioxidant	[51]
*Porphyra yezoensis*	Ultrasonic bath; 20; 100, 200 and 300	10	200 (water)	20, 40 and 60	15, 30 and 45	Taurine	-	[55]

**Table 3:** Subcritical Water Extraction (SWE) of bioactive compounds from marine macroalgae and their bioactivity.

Macroalgae Species	Ultrasound Operating Conditions					Bioactive Compounds	Bioactivity	Ref.
	Sample Mass [g]	Water Volume [mL]	Pressure [MPa]	Temp. [°C]	Time [min]			
*Sargassum muticum*	2	-	10.3 (1500 psi)	50, 125 and 200	20	Polyphenols, phlorotannins	Antioxidant	[34]
*Padina pavonica*	0.65	-	15	60	10 (2 cycles)	-	Anti-hyaluronidase	[58]
*Fucus serratus, Laminaria digitata, Gracilaria gracilis, Codium fragile*	2.5	-	10.3 (1500 psi)	120	25	Polyphenols	Antioxidant	[64]
*Cystoseira abies-marina, Porphyra* spp.*, Sargassum vulgare, Sargassum muticum, Undaria pinnatifida, Halopitys incurvus*	1	-	10.3 (1500 psi)	120 and 200	20	Polyphenols, neo-antioxidants, amino acids	Antioxidant and anti-microbial	[69]
*Himanthalia elongata*	1	-	10.3 (1500 psi)	100	20	Poly-saccharides	Antiviral	[14]
*Saccharina japonica*	9.65	160	10	150	5	Fucoidan	Antioxidant	[12]
*Saccharina japonica*	6	150	1.3–52	180–420	5	Total organic carbon, minerals, amino acids, mono-saccharides	-	[70]
*Ascophyllum nodosum, Pelvetia canaliculata, Fucus spiralis, Ulva intestinalis*	2.5	-	10.3 (1500 psi)	120	-	Polyphenols	Antioxidant	[66]
*Saccharina japonica*	5	160	5	100–250	5	Polyphenols	Antioxidant	[67]

**Table 4:** Microwave-Assisted Extraction (MAE) of bioactive compounds from marine macroalgae and their bioactivity.

Macroalgae Species	Ultrasound Operating Conditions				Bioactive Compounds	Bioactivity	Ref.
	Power [w]; Frequency [MHz]	Solvent	Temp. [°C]	Time [min]			
*Padina pavonica*	1000; 2450	petroleum ether, ethanol, ethyl acetate and H_2_O	40, 60, 80, 100 and 120	2 and 5	-	Anti-hyaluronidase	[58]
*Caulerpa racemosa*	100–600; -	20–100% ethanol	20–70	5–60	Polyphenols	Antioxidant	[60]
*Enteromorpha prolifera*	300–700; -	10–60% ethanol	-	5–40 (1–4 cycles)	Polyphenols	-	[76]
*Carpophyllum flexuosum*	-	H_2_O, acetone, ethanol, propan-1-ol, ethyl acetate	135, 160 and 185	1, 3, 5, 10, 15 and 20	Phloroglucinol	-	[74]
*Undaria pinnatifida*	600; -	H_2_O	110–120	5–120	Fucoidan	-	[79]
*Sargassum thunbergii*	200–800; -	H_2_O	10–90	10–50	Poly-saccharides	Antioxidant and hypoglycemic	[75]
*Fucus vesiculosus*	-	H_2_O	122, 152 and 172	1, 16 and 31	Poly-saccharides (fucoidan)	-	[78]
*Ulva meridionalis, Ulva ohnoi, Monostroma latissimum*	1000; 2450	H_2_O	100–180	10	Poly-saccharides (ulvan and rhamnan sulfate)	-	[83]
*Ascophyllum nodosum*	-	0.1 M HCl	90, 120 and 150	5, 15 and 30	Fucoidan	Antioxidant	[81]
*Ulva prolifera*	500; 2450	0.1 M HCl	90, 120 and 150	15	Poly-saccharides	Antioxidant, anti-hyperlipidemic	[82]
*Carpophyllum flexuosum, Carpophyllum plumosum, Ecklonia radiata*	-	H_2_O	160	3	Phlorotannins	Antioxidant	[77]
